# Dimensions, Dialectic, Discourse. Three Political Perspectives on the Sustainability of the German Healthcare System

**DOI:** 10.3390/ijerph15071526

**Published:** 2018-07-19

**Authors:** Matthias Fischer, Harald Heinrichs

**Affiliations:** 1 Comparative Politics and German Government, Institute of Political Science and Sociology, University of Würzburg, Wittelsbacherplatz 1, 97074 Würzburg, Germany; 2 Sustainability and Politics, Institute of Sustainability Governance, Leuphana University of Lüneburg, Universitätsallee 1, 21335 Lüneburg, Germany; harald.heinrichs@uni.leuphana.de

**Keywords:** sustainability, German healthcare system, policy, politics, polity

## Abstract

This review article deals with the topic of sustainability in the German healthcare system and presents an overview of how the six articles of our research relate to one another. After introducing to the context of the research, its internal principles, and the methods applied, three perspectives are presented, each also discussed in terms of the respective literature in sustainability science and political science. The review concludes by presenting a circular model and by discussing the general limitations as well as the practical implications of our research.

## 1. Introduction

The German healthcare system is facing some immense structural challenges, despite its considerable and 150-year lasting continuity [1]. This refers to—just to mention some of them—demographic challenges and questions of how to guarantee long-term financing and inter-generational justice; or the possibilities and obstacles of digitization, for instance, in light of the opportunities and challenges of modern health information technology [2,3,4]. However, these and other challenges seem to be general and structural problems, which many industrialized societies are being confronted with—regardless of the concrete differences in terms of how healthcare is provided, regulated, or financed [5,6]. ‘Sustainable development’ or ‘sustainability’, defined as “development that meets the needs of the present without compromising the ability of future generations to meet their own needs” [7] (p. 41) by the World Commission on Environment and Development (WCED) report from 1987, could be an attractive goal for healthcare systems. However, instead of a sustainable development within the European healthcare systems, one is tempted to speak of a “reversed sustainable development”; while the challenges exerting pressure on the healthcare systems of the industrial countries seem to grow ‘sustainably’, the institutional structures do not seem to adjust or ‘develop’ accordingly. Starting with these challenges in mind, our research has been dealing with the broad topic of ‘sustainability and the German healthcare system’, thereby focusing on the regulatory-political framework of the healthcare system, over a period of more than four years.

Within this broad scope, the research also addressed three specific perspectives, which are held together by two narrative brackets. Table 1 gives an overview of the two narrative brackets, which structure the perspectives and arrange the specific articles. The first narrative bracket consists of the three dimensions of the political term. While ‘polity’ refers to political structures and institutions, ‘politics’ describes the struggle for power among and between the political players and the general process of policy-making, whose actual content and results, in the form of laws or regulations, are understood as ‘policy’ [8]. Although the terms might be used very intuitively by English native speakers, their different scopes provide for clarity with regard to their meanings, making them a suitable tool to structure our research. Table 1 demonstrates that all three dimensions of the political term have been addressed by the articles (see also Section 3 of this review), even though each of them could be explored more in depth and breadth by future research.

- Firstly, it was seen as a necessary question to elaborate further on what is meant by ‘sustainability’ when it is applied in the context of healthcare systems and health policy. This clarification was considered as especially important for the context of healthcare systems as—particularly with regard to the German social state—the notion of sustainability still faces prejudices and misunderstandings.
- Secondly, our research asked how sustainability can unfold its potential in concrete health politics (i.e., the daily struggles of a diverse set of players for power and influence). The goal was to take a deeper look into the ideological roots of the system in order to understand these struggles better.
- And thirdly, as a consequence of the analysis of the system’s roots, the question arose as to whether it is possible to design an institutional environment, a polity, which is able to enhance the potential of sustainability within the healthcare system in terms of sustainability-related factors (e.g., transparency or participation).

The second narrative bracket can be described as the advancement concerning methodological innovativeness as the research proceeded. While the research began with the elaboration of a framework for sustainability in healthcare systems based on a conventional literature review, its second part consisted of an in-depth analysis of the ideological roots of the healthcare system by applying grounded theory methodology, which was less regulated by the methodological literature and gave us more freedom in deriving the theoretical concepts [14]. The third part presented the results of a design thinking study. Design thinking belongs to the most recent approaches of sustainability science and is explicitly meant to open the solution space very broadly [11]. Thus, one could say that the research started with traditional means but then gradually entered deeper into unconventional and innovative areas of scientific methodology.

## 2. Materials and Methods

### 2.1. Literature Review

Starting with a short review of the history of the term ‘sustainability’ on a general basis, a literature review was initiated to analyze what the relevant literature understands by ‘sustainability in the healthcare system’ or a ‘sustainable healthcare system’. The goal of the first perspective of our research was not to present a holistic and deep-level illustration of every existing piece of research. It was rather supposed to give an overview of past approaches, to deduce a synthesizing state-of-the-art framework based on the most significant examples in the aftermath. In order to structure the literature review, relevant approaches were checked for different parameters, such as to whether they made explicit reference to the WCED report or whether their focus was on a single healthcare system or on a cross-country comparison.

### 2.2. Grounded Theory Methodology

The motivation of the second perspective of our research [9,10] was to have a closer look at the foundations of the German healthcare system by applying grounded theory methodology (GTM). The openness and the nature of GTM as an emergent method has the advantage that the researcher may adapt it flexibly to his or her specific needs [15]. For the purpose of our research, a total of 14 interviews with top-level health–political representatives was conducted (for a detailed description of the questions, see Appendix A of the article [9]). The interview partners were recruited based on the principle of theoretical sampling, in which the researchers assess potential research directions depending on the outcomes of the analysis of an interview, and select the following interview partners based on their suitability to contribute to the new direction [16]. We considered this approach as superior in comparison to, for example, the a priori determination of the sample. The coding procedure was adapted from Glaser [17], and Glaser and Strauss [18], and followed a two-step process. A high number of descriptive codes were derived out of the data in the so-called ‘substantive coding’, before these relatively concrete codes were transferred into abstract theoretical concepts in the so-called ‘theoretical coding’ [17]. This made it possible to read and interpret the text and impressions through the lens of the emerging theory. Throughout the whole analysis, memos were drafted to capture the thoughts and intermediary results of how the derived concepts could interrelate.

As the research proceeded, the analysis focused more and more on the existing contradictions and dependencies among the stakeholders of the healthcare system. Within this stage, the data was enriched with some of Georg Wilhelm Friedrich Hegel’s thoughts on the dialectic of modern bourgeois societies. Furthermore, the elaboration of the dialectic was seen as an opportunity to raise a voice in the discussion of whether welfare in modern high-income countries can be thought of with or without growth [10]. Of course, one might critically discuss the benefit of including both Hegel’s thoughts and the connection of the data with the growth-welfare debate, when approaching the healthcare system by itself is already a highly complex matter. Our argument would be that dealing exclusively with the technical question of how to improve the German health governance would have not sufficiently addressed the tremendous systemic challenges of the healthcare system. As the German healthcare system is embedded and plays a significant role in the growth-dependent German economy, it was seen as legitimate to include literature dating back to the foundational years of Germany’s industrialization, in order to answer fundamental issues beyond the surface level.

### 2.3. Design Thinking

In order to endow the research with a look ahead, our third perspective [11,12,13] applied design thinking with its intuitive procedure and its bias towards action, making its application feasible in spite of limited personal and financial resources. Design thinking suggests starting the research with the thorough analysis of the respective research field [19]. The first goal is to approach the respective field of interest very generally in order to ‘understand’ how the respective stakeholders perceive their environment and how they deal with structural limitations. The researcher should develop empathy for the players in the field. Afterwards, a concise problem statement defines the actual ‘wicked problem’ of the research. The problem statement is a qualitative and subjective decision of the researcher, which is the starting point for the creative flow of ideas. This brainstorming aims to ideate as many ideas as possible. Afterwards, design thinking suggests transferring some ideas to the real world by ‘prototyping’ low-cost preliminary artifacts of the ideas. These prototypes serve as the basis for gathering feedback in the ‘test’ mode, and initiating new cycles of idea-creation [19,20]. A table with the detailed description of how our research approached each of these modes is described in the article by Fischer [12].

As design thinking encourages collaborative work [19], we drew upon the expertise and ideas of workshops with students and colleagues during several points of the design thinking research. One could critically ask whether the design thinking study should have been conducted with a broader sample of more representative stakeholders of German health politics. Certainly, with the selected sample of contributors, biases in terms of possible alternative ideas cannot be excluded. However, instead of providing for an exhaustive study taking into account all possible healthcare stakeholders, our research was supposed to serve as an experimental pilot study, as an exploratory test of the new methodology of design thinking, using the example of German health politics. By including the expertise of the non-representative sample, the Virtual Reality (VR) Health Arena was developed as one out of potentially many other ideas.

## 3. Policy, Politics, Polity: Three Perspectives on the German Healthcare System

This section presents each of the three perspectives in greater detail. The results of each perspective are summarized, before each perspective is discussed, with regards to the respective literature of both political and sustainability science. As the previous section has mentioned, our research drew upon methods whose range can be described as an increase in terms of methodological innovativeness. Because of this, the size of the following sub-sections varies as well. While the results of the conventional literature review can be described shortly, the more innovative approaches were supposed to receive a more detailed discussion.

### 3.1. Policy: Defining Decisive Factors of Sustainability in Healthcare Systems

#### 3.1.1. Description of the Main Outcomes

Based on a review of the existing literature around the topic of sustainable healthcare systems, the first perspective with the article “Fit for the future? A new approach in the debate about what makes healthcare systems really sustainable” [6] has discussed the various interpretations of sustainability in the context of healthcare systems. However, despite the whole variation, most of the approaches seemed to apply a comprehensive approach, which targets a balance of economic and socio-ecological interests, and suggests addressing needs with a strategic long-term perspective. Based on this basic consensus among the literature, we condensed the findings of the review to five categories of a new and innovative state-of-the-art framework. These categories were suggested as major assets of sustainability in healthcare systems, and could also serve as guiding principles for health policy-makers, namely: (1) a long-term strategic perspective and the promotion of innovativeness; (2) disease prevention and health promotion; (3) a focus on quality; (4) the institutionalization of environmental concerns, with the environment covering both ecological concerns as well as broader health determinants, taking into account the systemic interrelations of health with other policy fields; (5) as well as institutional accountability and individual responsibility, which would include the enhancement of participatory elements within the regulatory framework of the healthcare system.

#### 3.1.2. Scientific Context

The approach of the framework fits into longstanding attempts in the sphere of politics and political science to align policies with distinct, desirable factors in order to guide them towards certain directions, including the development of health performance indicators. For example, beginning in the year 2000, there has been an attempt in German health politics to define ‘health targets’. Resulting from a cooperative discourse of different healthcare stakeholders and scientists, the targets were supposed to be the starting point for improving the overall health status of the population, inter alia, by initiating several health-related projects and by monitoring their societal impact. However, despite the fact that, in 2017, a new target was added with a focus on “health around birth”, the project was partially criticized, as the approach still seems to lack the consequent integration of the targets across the different federal states [21].

Also, on a global level, health indicators aiming to measure the performance of healthcare systems and the general health status of populations have been defined, promoted by international organizations such as the World Health Organization (WHO) or the Organisation for Economic Co-operation and Development (OECD) [22,23]. Within the set of indicators, analyses on a meta-level have shown that ‘sustainability’ is—if it is mentioned at all—only one desirable parameter among many others [24]. Frankly speaking, sustainability has not become the overall guiding principle for assessing the performance of healthcare systems. In searching for the reasons for this, one could refer to the high degree of abstraction, where sustainability might fall behind more concrete and potentially easier measurable concepts, such as efficiency or accessibility [25]. Furthermore, when taking a closer look at the concrete application of ‘sustainability’ in the analyses, one gets the impression that sustainability is often equated with ‘macroeconomic efficiency’ [24] (p. 7); thus, as a synonym for sustaining the existing structures and keeping the costs of a system under control. However, according to the review in Fischer [6], sustainability includes many more aspects than maintaining the status quo. Additionally, these health-related indicators are often applied within a certain context when they, for instance, address a country’s development regarding certain health risks or in relation to occupation-based indexes (e.g., the ratio of physicians related to the population) [23].

**Figure 1:**
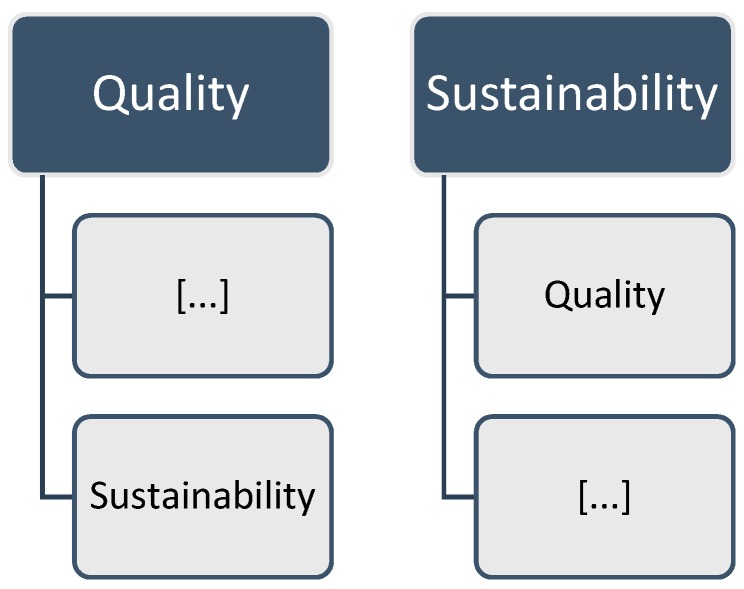
In conventional quality indexes, sustainability is (if mentioned at all) only one among other indicators (on the **left**); Fischer [6], on the contrary, puts sustainability in the center (on the **right**)—without decreasing its importance, quality is defined as one among other sustainability-related values (own illustration).

In consideration of these thoughts, Fischer [6] put sustainability as a desirable value for the healthcare system as a whole in the center of the analysis, instead of a focus on ‘quality’ or other values of the common performance indicators (Figure 1). With this procedure, the article hoped to position the concept of sustainability more prominently as the central goal for health policy-makers. At the same time, in light of the wide variety of understandings and conceptualizations in terms of ‘sustainability’ and other performance indicators, it tried to incorporate the most important aspects of ‘good’ health policy under the imaginary umbrella of sustainability, in order to define a common ground. Therefore, ‘quality orientation’ was defined as an important determinant for the sustainability of a healthcare system, as well as further essential factors (e.g., a long-term strategic perspective, health promotion, or the institutionalization of environmental concerns) for improving the performance of the healthcare system. In order to counter the challenges, as mentioned in the review’s introduction, the overall framework suggested a “comprehensive approach with a long-term focus and a need to balance economic, social, and ecological interests” [6] (p. 298). We believe that the five factors of the framework could serve health politicians in estimating potentials for promoting sustainability in the healthcare system.

In addition to the contribution to the literature on performance indicators in political science, the framework can be said to have incorporated central aspects of ‘sustainability’, which have been discussed in sustainability science. For instance, the factor ‘institutional accountability and individual responsibility’, in Fischer [6], made the suggestion that after their theoretical deduction by the author and before their final manifestation, the five factors should be discussed by the civil society in a social discourse on the preferences and priorities for policy-makers. This demand for participation and cooperation, which has become the core contents of the second and the third perspective of the review, met one of the central claims of discussions on how a ‘sustainable society’ could and should be organized [26]. Additionally, the importance of institutional transparency, as well as the systematic and broad inclusion of environmental concerns in the discussion of health-related sustainability, have emphasized the general importance of understanding health as a complex interplay of various factors [23,27].

### 3.2. Politics: Health Politics and Its Underlying Dialectic

#### 3.2.1. Description of the Main Outcomes

Based on the interviews of the article by Fischer [9], health politics appeared to be a very intense struggle of the players, in which there is often a perceived separation between the winners and losers. In order to maximize their own share from the overall sum of money that is to be distributed, the stakeholders observed the actions of the other stakeholders carefully. The goal was to anticipate possible counterstrategies and seeking to prevent the respective opponent from receiving overly benign assessments, which would have been perceived as a potential threat to one’s own position. As a consequence of the mutual ‘sitting in ambush’, some interview partners described a constant fear of missing a decisive point in the struggle for money, resources, and influence. The main goal of each stakeholder was thus interpreted as the enforcement of one’s own position, even if it should happen at the expenses of others. However, the interviews also revealed that the players were well aware of the systemic character of the healthcare system with its multiple interdependencies between the different stakeholders. Some stakeholders showed a high awareness that they could achieve some of their goals only in joint actions with other stakeholders. The inclusion of the respective opponent had required them to check for opportunities, which would also allow the included counterpart to benefit from the mutual actions. Despite the mistrust between the stakeholders, caused by their intense competition, there seemed to be a basic consensus among the players that an effective health policy would invest in the general welfare of the society, even of this would imply the neglect of individual interests.

As a consequence, the concrete examples of contradictions described by the health–political stakeholders were abstracted and condensed to two basic ideas in the article “Welfare with or without growth? Potential lessons from the German healthcare system” [9]. The first basic idea, which was defined as an “exclusive basic idea”, described the relation to one’s self and the exclusive pursuit of one’s own benefit. On the other hand, the “inclusive basic idea” describes the relation to others and the quest for social contact. Both basic ideas were considered as contradictory and yet interdependent. Following from this abstract contradiction, the dialectic was expected to result in concrete contradictions within the German healthcare system. This led to a new definition of the healthcare system as “a system of needs related to health, which is constituted by a constant contradiction between an inclusive and an exclusive basic idea” [9] (p. 6). As a forward-looking possibility to balance the contradictions caused by the two basic ideas, the first author suggested ‘sustainability’, under whose imaginary umbrella societal value discussions could be held within the current sociopolitical framework. These institutionalized discourses could possibly lead to a mitigation between the two basic ideas, as it would be possible to filter out contents that are capable of consensus, and to use the citizens’ participation as the basis for prioritizing the most urgent problems of the healthcare system. Thus, the notion of sustainability would imply a procedural understanding of the concept [26].

As described in Section 2, the data of the study was enriched with distinct parts of Hegel’s dialectic, after the importance of contradictions within the healthcare system had become apparent. This was supposed to discuss the embeddedness of the healthcare system within the broader context of the growth-dependent German economy, under the slightly ironic title “The growth-welfare dialectic: What might Hegel say” [10], by introducing a total of four theses. The first thesis suggested approaching the emotionalized growth-welfare debate with a reserved language. Even though problems related to the dependency of modern Western economies on growth may require radical responses, leading the debate with a tone of moral supremacy might cause adverse effects, as this could hinder a differential debate. The second thesis argued that, given the existing socio-economic structures, growth should not be treated as if it could be replaced like an instrument. On the contrary, a discourse on the ‘kind’ of growth a society aims for was suggested as being more promising. In the third thesis, the historic interrelatedness between growth and welfare in Western industrialized societies was discussed. It was argued that the quest for economic success has frequently gone hand in hand with benign actions for the society. Growth should, thus, be seen as both the basis for and a consequence of social welfare. By giving distinct examples, the first author considered that this was especially valid for the healthcare system. Based on the other three theses, and somewhat emphasizing the conclusions of Fischer [9], the fourth thesis suggested ‘sustainability’ with its core values of transparency and participation as an attractive concept for mitigating the debate.

#### 3.2.2. Scientific Context

The research interest of the second perspective of our research was triggered because of the perceived lack in the relevant literature regarding the ideological roots of the healthcare system, as research on the sustainability of the healthcare system certainly relies on hands-on reform approaches (e.g., [28]) and cost-effectiveness analyses (e.g., [29]). The differentiation between the contradictory concepts and dichotomizations can be found in several pieces of political and sustainability science. In the following, three kinds of research in political science shall be discussed—also in terms of their differences with the approach of the two basic ideas. Afterwards, the basic ideas will be placed within three perspectives of sustainability science, and will then be discussed in terms of a concrete example from the notion of sustainability, applied in the context of health politics.

Firstly, one could discuss the approach of the two dialectic basic ideas in relation to the politico-sociological opus of Bourdieu and his notion of “social fields”, in which actors and institutions engage in struggles for power [30]. The direction of public policies is assumed to be determined by the power of disposition regarding specific resources within these conflicts, which consist of different and partially substitutable forms of capital [31]. For example, one could imagine that actors invest their social or cultural capital consciously, in order to increase their economic capital. The specific allocations of the different forms of capital are assumed to affect the capability of a field’s players to enforce decisions. According to the theory, the dominant actors use their capital in order to impose the dominant interpretative patterns of a field upon the other actors [32]. However, the relationships among the players are not stable, but rather constant “struggle[s] for the production and imposition of the legitimate vision of the social world” [33] (p. 22). Compared to Bourdieu’s approach, the ‘basic ideas’ do not refer to specific resources and allocations of power. Even though the approach of the dialectic assumes a conflict-laden social field, in which different actors constantly strive for dominance, it is more about (so to speak ‘unloaded’) action patterns that are observable through the behavior of the actors and the respective policy outcomes. Thus, the dialectic looks closer on the way in which, for example, the resources of power are applied, namely in a more including or excluding sense. It also looks at the difference these action patterns make in terms of different policies, instead of a precise analysis of the invested resources themselves. As a second difference, the theory of the social field assumes the ignorance or lack of awareness of the players concerning the historical allocation of capital [34]. Contrary to this, the players in German health politics described their application of inclusive and exclusive action patterns as a very conscious decision.

Secondly, a distinct example of contradictions between economic and social imperatives within the literature of political science is Okun’s [35] elaboration of a goal conflict, which he defined as an equity–efficiency trade-off. Okun argued that the trade-off is caused by the phenomenon that the quest for far-reaching economic and social equity, which is generally the normative aspiration of social policy, interferes with the goal of economic efficiency. While Okun based his assumptions on the outcomes of economic activity, a third kind of contribution in political science has described contradictions specifically in terms of different patterns of policy-making. For example, Gerhard Lehmbruch differentiated between “concordance-democratic” processes, which are guided by the maxim of a mutually “amicable agreement” [36] (p. 208) between all of the involved stakeholders, as opposed to the “competitive democracy”. The latter is characterized by the principle of competitive policy-making, implying the subordination of policy-making under the majority’s will [37]. Compared to both approaches (the equity–efficiency trade-off and the different patterns of policy-making), one could argue that the two basic ideas would pursue a more holistic approach. In contrast to the equity–efficiency trade-off, which mainly describes differences in the observable outputs of market economies (i.e., social equity versus economic efficiency), and in addition to attributing the processes of policy-making to a more consensual or competitive pattern of democracy, the approach of the two basic ideas would presuppose that the presence of contradictions are inherent in both. This means that, on the one hand, social and economic ‘outcomes’ could be attributed to one of the basic ideas. However, the basic ideas would already interpret the process of getting to these outcomes as a dialectic struggle of actors behaving either more self-centeredly or more relatably to others. Both approaches could thus be subsumed under the dialectic.

If one brings sustainability science into focus, one would probably not assume the promotion of contradictions and dichotomizations; even less so, as sustainable development has been literally understood as a “process of change in which the exploitation of resources, the direction of investments, the orientation of technological development, and institutional change are all in harmony and enhance both current and future potential to meet human needs and aspirations” [7] (p. 43). This is why general conceptualizations of sustainability aim to harmonize different sustainability-related aspects, whether by balancing economic, ecological, and social pillars of sustainability, or by an integrative sustainability approach, in which cross-sectional terms are defined in order to approach multiple dimensions at the same time [38]. However, despite these general tendencies of harmony, the supremacy of the socio-ecological dimension in the case of conflicts between different imperatives has been frequently promoted. For example, Khan [39] postulated always putting social achievements ahead of the economy in a growth-critical article. Alternative examples are provided by the literature on de-growth and post-growth, which has interpreted the quest for economic success as one of the main causes for the global sustainability challenges. Consequently, this literature has promoted alternative ways of living, which would put humanist and solidary values at their center [40,41]. These approaches’ strong claim for putting social needs generally ahead of economic imperatives prompted the first author to add a differential voice to the debate on whether welfare could be achieved with or without at least respecting growth imperatives, based on the approach of the basic ideas. Both articles [9,10] argued that the pursuit of growth under the given socio-economic conditions needs to be understood as an expression of the idea of individual liberty. Based on this premise, the suggestion was not to question growth by all means, but rather to ask which kind of growth should be striven for under which conditions. With these premises, the articles could be attributed closely to the literature on green growth [42].

Looking at a second kind of research in sustainability science, one could argue that there are at least some similarities in the literature on the so-called weak and strong sustainability [43,44] in terms of the different focus of each basic idea. Weak and strong sustainability have discussed the possibility of substituting the environment by simultaneously enhancing the economy [45]. Weak sustainability would argue that solutions affecting the natural environment would be sustainable if natural capital is balanced by the production of other “artificial” [43] or “manufactured” [46] forms of capital. Simply speaking, according to this approach, an economically efficient performance could be sustainable if it increased the overall wealth of a society. On the other hand, according to the argumentation of strong sustainability, natural capital, which consists of natural resources and units of cultural significance, is complementary to artificial capital [43]. Acknowledging the complex systemic interrelationships of nature, this means that it can only sometimes be substituted and thus should be conserved by all possible means and over time [46]. Because of its basic differentiation between contrasting alternatives, one could argue that the approach of strong and weak sustainability has some common ground with the dialectic basic ideas. Just as it seems legitimate from the perspective of weak sustainability to increase the overall wealth by contributing to economic progress, the exclusive basic idea in its purest form would see the pursuit of growth as a deeply embedded driving force inside of each individual. Both the exclusive basic idea and the concept of weak sustainability consider it as justifiable to increase capital to maximize the overall utility, regardless of the consequences for the (social or ecological) environment. One might argue that the exclusive basic idea bases the contradiction on another counterpart, as its logic refers to the struggle of the individual advancing his or her own standing at the expense of other stakeholder(s) within a ‘social’ system, whereas weak sustainability refers to the question of whether economic advancement can balance losses in terms of ‘ecological’ capital. However, the boundary that separates the individual from its counterpart (i.e., the ‘other stakeholder’) in the dialectic, could also be adapted towards the demarcation of the human society in maximizing its profits at the expense of the natural environment. In the case of a specification towards this direction, the justification of the exclusive basic idea would be quite close to the assumption of weak sustainability, that environmental damages might be tolerable if only the overall capital is increased. On the other hand, the inclusive basic idea with its systemic view and the quest for satisfying the needs of the other stakeholders would have an intellectual affinity for the concept of strong sustainability. The inclusive basic idea is characterized by an altruistic aspiration for maximizing the collective utility, which could also imply the inclusion of the natural foundations of a society. Because of these similarities—and despite the aforementioned difference in terms of the focus on the ‘ecological’ pillar (strong/weak sustainability) as opposed to the emphasis of the abstract basic ideas on the trade-offs between actors and their respective ‘social’ system—one could see the distinction between strong and weak sustainability as somewhat of a concrete manifestation of the two abstract basic ideas.

**Figure 2:**
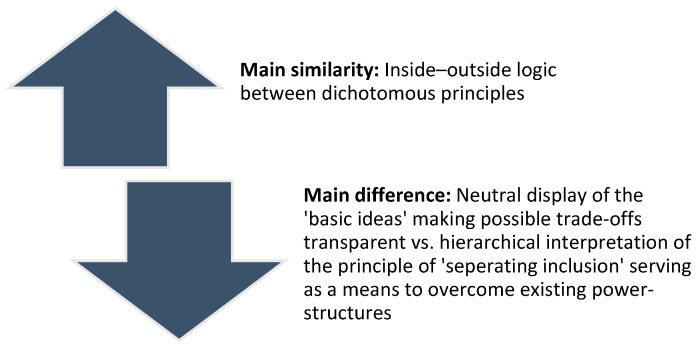
Comparison of the major similarity and difference between the basic ideas and the principle of separating inclusion (own illustration).

As a third perspective of the sustainability scientific literature, one could refer to the example of Biesecker and von Winterfeld [47] from the feminist literature on the topic of gender justice and gender equality. Biesecker and von Winterfeld argue that the progression of capitalism has been falling together with a continuous inclusion and exclusion of, inter alia, certain social groups. Furthermore, the authors argue that the excluded part has implicitly served as a means to stabilize existing power structures (i.e., the dominant capitalist system). According to their argument, this pattern has historically led to disadvantages, especially for women. The authors define the foundation of this “dialectical contradiction” [47] (p. 14) as “specific pattern of inclusion: The separating inclusion” [47] (p. 14). Not even the equal integration of women in the labor market would improve the situation, as the authors expect that this would not be followed by an equal number of men in the care professions in exchange (i.e., in non- or under-paid work in which women are overrepresented anyway). Defining this kind of externalization as a principle and not just as ‘externalization’ in an exclusively neoclassical economic sense [48] could be seen as a similarity between the basic ideas and separating inclusion, as both approach their arguments with an inside–outside logic. However, both approaches also show some significant differences (Figure 2). The approach of ‘separating inclusion’ by Biesecker and von Winterfeld should not just be understood as the description of contradictory and yet interdependent dichotomizations responsible for societal dynamics, just as the approach of the basic ideas. Their approach combines the neutral description of the principle with a hierarchical component, as the principle of separating inclusion served the dominating social groups (i.e., mostly men) as a means to exert power and should be overcome. This normative aspiration of overcoming existing power structures is not inherent to the basic ideas. The dialectic between the exclusive and inclusive basic ideas was neither judged as negative or as an impediment preventing social progress. The neutral explanation of the two basic ideas aims at making possible trade-offs transparent. At least within the current structures, neither pursuing a purely exclusive approach nor an inclusive approach of serving only the benefit of others would be desirable. The dialectic basic ideas thus were not seen as a problem to be overcome, but rather as system-immanent and mutually interdependent.

Based on this argument, the relativity of sustainability concepts in terms of their concrete contents could be shown in an altogether different light. For instance, an understanding of sustainability, which would effectively describe a long-term sustaining of economic success, could be seen as an exclusive approach towards sustainability, as it would tolerate the exclusion of the socio-ecological environment and the neglect of social value creation. As opposed to this, an understanding of sustainability, which would neglect economic imperatives and exclusively put the socio-ecological value creation at its center, could be defined as an inclusive approach towards sustainability, implying the threat of being exploited by freeloaders. It is highly probable that in reality, both basic ideas will not be found in their purest form in concrete conceptualizations of sustainability. We would argue that a comprehensive and differential understanding of sustainability would imply the mitigation of both basic ideas by a fair and structured social discourse. In such a discourse, both basic ideas and concrete manifestations of the ideas would have their justified place.

In order to illustrate this, a concrete example could be taken from the context of German health politics. In the discussion on reforming the financing of the German healthcare system based on the suggestions of the commission “Sustainability in Financing the Social Security Systems”, two major reform ideas were introduced, namely: On the one hand, the so-called “all citizens’ health insurance”, promoted by left-wing political parties in different nuances, suggested the inclusion of stakeholder groups that had hitherto not contributed to the community of solidarity (i.e., the compulsory health insurance). On the other hand, the so-called ‘health premium’ in its original form, planned for the introduction of a fixed individual contribution for each citizen, without differentiating between contributors in terms of their income or social status [49]. Both concepts have been explicitly promoted as ‘sustainable’ solutions and shaped the political debates for years [50,51]. The benefit of the dialectical approach of our research would be to provide for a higher degree of transparency. The goal would be to argue in favor of or against the ‘sustainability’ of each of the two concepts, based on a more informed decision. For example, one could argue that both the ‘all citizens’ health insurance’, with its focus on social solidarity, and the ‘health premium’, stressing individual responsibility in order to ensure long-term financing of the German healthcare system, could be considered as ‘sustainable’. When an exclusive approach is applied, the ‘health premium’ would be more sustainable. In the case of an inclusive understanding, the ‘all citizens’ health insurance’ could be seen as rather sustainable. However, a comprehensive understanding of sustainability would have demanded a social discourse by the population. For the future, this would require politicians and academic scholars to create a forum of discourse, taking into account the arguments of both concepts, in order to find a balanced solution displaying the collective preference among the citizens afterwards. The development of the concepts on how such a discourse could be organized was discussed in the third perspective of our research.

### 3.3. Polity: Designing the Discourse on Health-Related Values

#### 3.3.1. Description of the Main Outcomes

After the necessity of societal value discussions had become apparent in the second perspective, the third perspective was motivated by the question of whether sustainability challenges could potentially be approached as design tasks. As a preparatory step, the possible match between design thinking and sustainability science was discussed in the article “Design it! Solving sustainability problems by applying design thinking” [11]. After presenting the general characteristics of both sustainability science and design thinking, the article made its argument with a total of four theses. Firstly, it was suggested that both design thinking and sustainability science should develop applicable solutions for socio-ecological challenges, based on a systemic view. While sustainability science can already be characterized by a holistic world view, taking the multiple interdependencies of sustainability challenges into account [38], design thinking has thus far applied a rather narrow view based on individual customers, even though the first systemic attempts of design thinking could already be exemplified (e.g., [52]). The second thesis suggested striving for simple but not simplistic solutions, without neglecting the complex background of design or sustainability challenges. The other two theses referred to the possibility of bringing both design thinking and sustainability science closer together; the third thesis argued that both design thinking and sustainability science have a common ground in applying a positive idea of man, which can shape and improve the conditions for general well-being. The fourth thesis described that both design thinking and sustainability strive for disruptive breakthrough solutions in order to make a difference in the world.

As a concrete result of this conclusion, the article “The future of health debates? A design thinking sketch of the VR Health Arena” [12] presented the outcomes of approaching German health politics by means of design thinking. After focusing on potential means to change the institutional structures, the deeper analysis of the field led us to the conclusion that the complexity of health matters hinders their discussion in a broader public discourse, even though this discourse would be crucial in order to identify the value preferences of the citizens. Therefore, the ideation sessions searched for participatory and accessible means to counter the insufficient discussion of health-political matters by the citizens. The eventual concept suggested that such a discourse should adapt to the media consumption patterns of the population; consequently, the vision of the ‘Virtual Reality (VR) Health Arena’ describes an entertaining show format in which teams would discuss health-related ethical and political matters exemplarily in a competitive setting. The audience would have multiple opportunities to follow the show, either passively on the basis of conventional TV or streaming services, or actively by making use of VR technology, which would enable the respective users to become a direct part of the studio audience and have multiple means of participation during the show. For example, VR users could vote for the respective winning team, or even participate as ‘jokers’ with the direct possibility of discussion with the teams on the show. In order to enrich the possibilities of being part of the studio audience with an active-cooperative component, a ‘smaller’ version of the VR Health Arena was added to the concept, in which users could discuss the respective health-related matters completely virtually, with other users. It becomes clear that, with the VR Health Arena, a wide interpretation of ‘polity’, beyond an exclusive reference to political structures and institutions, was applied. As the application of design thinking implied an unconventional research process, which according to Owen [53] required the application of an alternative criteria of goodness, the VR Health Arena was subjected to a qualitative assessment in terms of the three criteria, “cultural fit”, “appropriateness”, and “effectiveness”. The assessment lead to the conclusion that the VR Health Arena carries the potential of being an entertaining platform for specific social discussions, even though its concrete implementation would still require advancements in terms of the necessary VR equipment and potential risk factors (e.g., data protection concerns, cyber sickness).

Rapid prototyping is seen as a central characteristic of design thinking, compared to conventional forms of brainstorming [20]. The early-stage development of real-world, ‘tangible’ artifacts is seen as the basis for better understanding the functioning of an idea, in order to specify the prototype based on feedback loops afterwards [19]. Therefore, the article “Decoding sustainability in the healthcare system. Teaching students how to problematize complex concepts” [13] gave further insights into how the prototypical test of the VR Health Arena, with students at the University of Würzburg, Germany, was carried out. The case study describes the implementation of the forum of discourse in a university seminar, where the first author has been instructing sophomore students in the subject ‘health politics’ for several years. The aspired learning outcome before the start of the course was to enhance the reflexive and argumentative competencies among the students in terms of the future development of the German healthcare system. In order to enhance the students’ argumentative competencies, and to gain further insights for the development of the VR Health Arena on an ‘offline’ basis (i.e., without the inclusion of the virtual elements), the first author reserved one day of his four-day seminar in Würzburg. After introducing the students to the notion and history of sustainability, they had the opportunity to discuss several health-related value conflicts (e.g., “pro/contra of assisted suicide”). A qualitative and ungraded ex-post assessment of the students left the conclusion that the arena of discourse had really enabled the students to acquire the aspired competencies. The insights of this didactic experiment helped the first author to refine the final concept of the VR Health Arena in the respective article [12] before it was published.

#### 3.3.2. Scientific Context

When locating the VR Health Arena within the literature of political science, one could start very generally. Based on the assumptions of systems theory, Korte [54] suggested dividing the political sphere into three arenas. While the ‘parliamentary arena’ focuses on the struggle for power between politicians based on the majority rule, the ‘administrative arena’ describes the non-hierarchical and often hidden bargaining processes between political and administrative actors. Standing somewhat outside and yet often mitigating between the other two arenas, the ‘public arena’ not only describes the relationship between politicians and the general public, but also the legitimization of public policies by stimulating social discourse. As the basic goal of the VR Health Arena was defined as the enhancement of public discussions on health-related matters among the German population and the long-term integration of the show as a systematic part of the broader German health–political structures, it could be attributed to the public arena.

When looking at its conceptual basis, one could discuss the VR Health Arena in the light of Habermas’ concept of deliberative democracy. Habermas [55] assumed that creating a public space in which political arguments and individual preferences could be exchanged by the citizens in a rational manner, would lead to the adoption of the best policies. The VR Health Arena has incorporated some of the main ideas of this concept, such as the idea of making arguments explicit and providing a public sphere in which a large audience is addressed. However, the VR Health Arena renounces that these discussions need to result in actual decision-making. This premise was regarded as especially important, as the political space in Germany tends to be generally very ‘politically loaded’, implying a general atmosphere where the public arena is abused by media, lobby, and political actors in order to gain competitive advantages in the struggle for power [54]. Thus, the VR Health Arena, if realized, would be considered to be an ‘indirect’ institutionalization of sustainability. It would aim to institutionalize sustainability-related values such as participation, transparency, and public discourse [26]. The benefit, compared to both the original theoretical concept of deliberative democracy and to alternative forms of identifying societal value preferences (e.g., [56]), can be seen in the incorporation of recent advancements in virtual reality technology. To formulate it in slightly exaggerated terms, one could interpret the VR Health Arena as a modern variant of deliberative democracy; as “deliberative democracy 2.0”, bringing people together in order to negotiate socially relevant health matters adapted to the technological progress and embedded in a playful environment.

**Figure 3:**
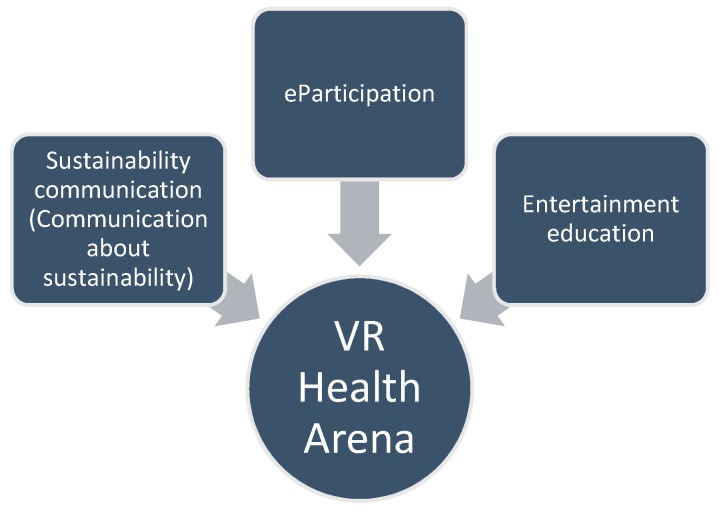
The concept of the Virtual Reality (VR) Health Arena could be seen in line with three streams of research on sustainability (own illustration).

The VR Health Arena was designed to be an explicit contribution to sustainable development and to make use of the possibilities of recent technological progress. One could see the VR Health Arena as located within several streams of existing research within the field of sustainability science [38]. Three of these connections shall be elaborated more concretely (Figure 3).

First, and most obvious, the connection to participatory approaches was already mentioned in the article [12]. Because of its innovative approach in terms of the applied technology, one could see a proximity of the concept, especially to the field of e-participation. In reviewing the contributions to this dynamically growing research field, one can observe that many of the approaches seem to focus on the technological means of implementing e-participation, which has resulted in the suggestion to take more of the contextual factors, such as the accessibility of the offers or political issues (e.g., the increase of transparency or the potential for opening the offers for new target groups), into consideration [57]. The vision of the VR Health Arena was designed to address the perceived need for making complicated health–political matters accessible for broader parts of the society. Consequently, the VR Health Arena would hope to make use of the opportunities connected with the recent technological advancements, in order to include broader parts of the society in political debates. This hope was seen as legitimate, as the ones who are most affected by health-related policy-making are the citizens themselves. By making the debate independent of power structures and majorities in parliament, while at the same time creating the polarized character of the discussions, people could experience the abstract values that are often behind health–political conflicts in a lively and entertaining manner—accessible via different channels.

With the motivation of enhancing the communication about sustainability-related content in health politics, one could classify the approach of the VR Health Arena, secondly, within the literature on ‘sustainability communication’. Newig et al. [58] presented an integrative framework for the practical and scientific orientation in this research area. The framework outlined three main directions of sustainability communication, differentiating between ‘communication of sustainability’, ‘communication about sustainability’, and ‘communication for sustainability’. While communication of sustainability can be described as a unidirectional sender–receiver flow of information for the purpose of education and information, communication about sustainability describes debates in which—often controversial—opinions and interpretations can be interchanged bilaterally between individuals [59]. On the other hand, regardless of the direction of the transported message and independent of who has initiated the discussion, communication for sustainability shifts the focus towards the explicitly normative goal of transforming the society towards sustainability. Within this spectrum of concepts related to sustainability communication, the VR Health Arena would target the stimulation of ‘communication about sustainability’, as it aims to create intersubjective exchange among and between the citizens, and to encourage the citizens to the development of empathy for the complexity behind concrete sustainability-related health policies. As the vision of the VR Health Arena, as mentioned, would make use of recent advancements in virtual reality technology, it would be in line with sustainability research, encouraging the utilization of new forms of communication for including broader parts of the society [60]. Furthermore, through the increased capability of reflecting on health–political matters, a discretion to act could be built within the society. This discretion could complement traditional, institution-based health politics with a growing pool of potential change agents from all layers of the society, thus contributing to the long-term goal of reaching the more transformative ‘communication for sustainability’.

With the target of contributing to the development of argumentative and reflexive competencies among the population by applying tools and practices of the entertainment sector, the VR Health Arena could, thirdly, be attributed to research on entertainment–education [61,62,63,64,65,66,67,68,69,70,71,72,73,74,75]. Entertainment–education can be defined as the “process of designing and implementing an entertainment program to increase audience members’ knowledge about a social issue, create more favorable attitudes, and change their overt behaviors regarding the social issue” [61] (p. 82). Another definition refers to entertainment–education as a “theory-based communication strategy for purposefully embedding educational and social issues in the creation, production, processing, and dissemination process of an entertainment program, in order to achieve desired individual, community, institutional, and societal changes among the intended media user populations” [62] (pp. 272–273). Both definitions point to the normative aspiration of changing the behavior of the target group of the entertainment offers towards an aspired direction.

Based on different publications and case-based examples over the years, there has been a growing body of evidence that entertainment–education seems to carry potential for raising awareness and triggering social change in certain fields [63,64,65]. Furthermore, the particular potential of the approach for addressing the specific trade-off situations concerning sustainability and in terms of enhancing social change for large audiences was emphasized [65,66]. However, facing the complexity of communicative processes, the literature has also suggested that entertainment–education may affect the behavior of the target audience only indirectly, for example, when it encourages participants to critically compare their individual attitudes with contradictory perspectives, or by enabling peer communication, social exchange, and interpersonal communication [63,66]. Especially serial formats, providing the respective audience with the opportunity to identify with the characters, which in the best case develop their personalities as the format proceeds, and the provision of the offers on multiple channels, were mentioned as additional success factors of entertainment–education [63,64]. The vision of the VR Health Arena could be seen as in line with some of its success factors, as it becomes clear by looking at three distinct features at the end of this section, as follows:

- *Stimulation of interpersonal discourse instead of unidirectional ‘education’ of the audience*: The approach of the VR Health Arena supports the voices of sustainability science in stressing that sustainability-related content should not be transmitted in the sense of top-down education [67]. It rather aims to confront people with different arguments, as it supposes that a non-hierarchical discourse is per se a desirable goal and a contribution to sustainable development.
- *Increasing the motivational attachment of the audience by cognitive/affective-emotional stimuli and the adjustment to contemporary media consumption patterns*: Instead of an exclusive focus on the presentation of the arguments in a rational manner, the VR Health Arena would try to reach the audience also on an affective-emotional level. In the best case, the play-off-based game mode of the show would lead to a continuous and intense engagement of the audience from different generations. The additional benefit of using VR technology would consist in its immersive potential, by giving the respective audience the impression of being directly part of the studio with the outlined opportunities to intervene.
- *Collective learning through the imitation of role models and the possibility to practice the argumentative and reflexive competencies actively*: The goal of the VR Health Arena would be to raise awareness for conflicting arguments in health–political matters, which are seen as constitutive for the healthcare system, in order to stimulate discussions among the audience. The respective teams would be coached in terms of their presentation style and develop their argumentative skills as the show proceeds. This personal development of the role models (i.e., the team members) could have a positive effect on the audience, as this could further strengthen the identification from the side of the audience. Furthermore, through the confidence of understanding the conflicting values in health-related matters better, as a result of their transparent comparison in the show setting, and by actively practicing the aspired competencies in the ‘smaller version’ of the VR Health Arena with other users, the feeling of self-efficacy among the audience could be promoted.

## 4. Conclusions

After the presentation and discussion of the three perspectives, the major conclusions shall be presented. Furthermore, some limitations of the research as a whole shall be discussed, in addition to the limitations already described in the specific articles. Last but not least, some practical implications of the research for the healthcare system and health politics shall be outlined.

### 4.1. Central Conclusions

This review is the result of more than four years of intense research on the regulatory-political framework of the German healthcare system. It should be evident at the end of this review that the research approached the three scientific issues that have been mentioned in the introductory section with a different set of methodological procedures, which have consequently lead to a variety in terms of the concrete outcomes. This review structured a total number of six articles by presenting each of the three perspectives’ main outcomes and placing the results within the literature on political and sustainability science. As was described in Section 1, the perspectives are thematically held together by the three dimensions of the political term and by their increase in terms of methodological innovativeness. Additionally, parts of the research allowed for looking beyond the field of health politics and raising a voice in broader discourses, such as the general adaptability of design thinking for the context of sustainability science [11], the question of whether social welfare can be thought of with or without economic growth [10], or the deeper discussion of how to design intangible prototypes for the future of health communication [13].

Based on this formal structure of our research, how could its main conclusions be beneficial for the discourse on the sustainability of the healthcare system? The first perspective of the research suggested five factors of sustainability in healthcare systems as an orientation for health politicians based on a literature review [6]. The factors were supposed to serve as an appropriate basis for deriving a set of measurable variables being proxies for healthcare sustainability in future research. At the end of our research and with the insights of the other articles—especially with their strong emphasis on transparent and participatory means of communicating health politics in the light of a dialectic between social and individual imperatives—one might suggest a more differentiated procedure for such a measurement. In addition to the expert review-based decision upon the most relevant categories for sustainability in the context of the healthcare system, future endeavors should find appropriate means to translate the complexity of health–political concepts to the main addressees of health–political actions, the citizens. Traditionally, the social scientific literature has treated expert knowledge and the outcomes of social discourse as highly different domains, supposing that both should be restricted to their specific area [68] or even assuming the incompatibility of their respective self-reproducing societal ‘codes’ [69]. Other scholars explicitly requested scientists to step down from the academic ‘ivory tower’ and to construct socially relevant knowledge [70]. In the light of this second direction, approaches of transdisciplinary research on sustainability have begun to consider the two forms of producing and constructing knowledge not as contrasting but rather as a complementary pair. For example, Renn [56] presented the “analytic-deliberative model”, in which he aimed to integrate the knowledge and value preferences of citizens into the scientific-analytic process of assessing future-related risks and co-developing possible counterstrategies. He proposed that analytical thinking and the deliberative exchange of arguments should not be treated as separate but rather as complementary components, both being important for a science-based cooperative process of policymaking. Taking into account the complexity and uncertainty of conducting science, the idea of approaches like this is to appreciate the usefulness of different forms of knowledge in their specific contexts, and to suggest ways to combine them effectively for the production of “socially robust”, contextualized knowledge for the benefit of both the academic and the practical world [70]. The role of researchers can be described as moderators, as they are expected to enable representatives of different forms of knowledge production to authentically introduce their perspectives and to map their normative preferences accordingly. Consequently, with its approach of combining expert knowledge on the notion of sustainability in the context of the healthcare system with the development of an innovative format (i.e., the VR Health Arena) in order to enhance the discussion of value preferences among the citizens, we considered neither expert judgments nor the integration of citizens’ beliefs as superior. Rather, its general approach and the derived concepts recommended to make use of both forms comprehensively in order to present scientifically sound [68], socially robust [70] knowledge, which includes potential benefits of the digital transformation [71]. The vision of our research is that in the future, health-related policymaking would be the result of both a societal discourse of informed citizens and the findings of scientific experts and possibly new insights from the digital world. With this ambitious goal, we would aim to establish a connection to a research area in political science, which has been defined as ‘policy design’. Whereas the conventional understanding of policy design had been restricted to the mere implementation of policies [72], the importance of a holistic application of design in structures and stages of the political arena was suggested more recently [73]. Of course, this vision of a vitalization of the political sphere by extending the means of participation and enhancing public discourse would require preparatory steps, such as a comprehensive educational strategy, which would also imply raising a high degree of awareness among the citizens regarding potential trade-offs between economic and social imperatives. A recent study concerning the health competencies of the German population, which is also known as ‘health literacy’, revealed that more than half of the German population seems to lack a sufficient degree of these competencies [74]. Based on the premises of our research, health competencies would not just refer to medically relevant knowledge and capabilities, but also include the argumentative and reflexive skills of the population concerning the health-related trade-offs, which have been described in the second perspective.

**Figure 4:**
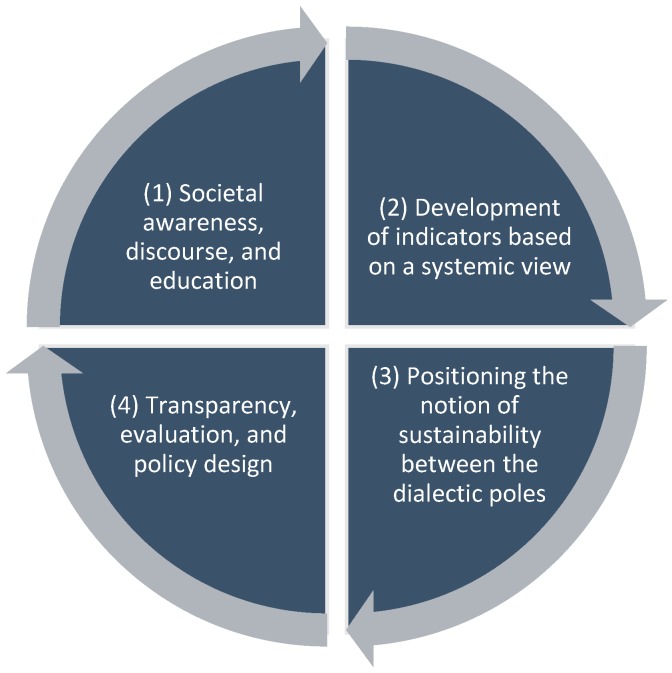
Circular model outlining the suggested procedure for a more differentiated look at the measurement of sustainability in the healthcare system (own illustration).

Thus, the final conclusions of this review could be combined to the circular model in Figure 4, which consists of four elements, namely:

- *(1) Societal Discourse, Awareness, and Education*: The enhancement of sustainability in the context of healthcare systems would imply increasing public awareness among the citizenry concerning the importance of discussing the values, different options, and future directions of German health policies. A display in terms of the preferences concerning the current and future form of the healthcare system could be achieved by strengthening the possibility for a number of citizens to participate in a social discourse. Design thinking could be a useful method for developing need-based policies with a strong participatory spirit, which should go hand in hand with a profound educational strategy, regarding the competencies of the population in terms of health and the healthcare system. In order to increase the health competencies of the population regarding their argumentative and reflexive capacities, the VR Health Arena could be both an entertaining and effective component of such a strategy.
- *(2) Development of Indicators based on a Systemic View*: Based on the discourse and the insights with regard to the population’s preferences, research on the sustainability of healthcare systems should still aim to define measurable factors of healthcare sustainability. These approaches would include a public discourse in which political, economic, scientific, and civil societal actors could participate. Such indexes of healthcare sustainability could also build upon previous attempts on the European and on the international level, with their strong interest of measuring the performance of healthcare system with relevant data (e.g., [75]).
- *(3) Positioning the Notion of Sustainability between the Dialectic Poles*: The results of our research would make it possible to come to a more conscious decision, as each reference to sustainability in the public discourse could be positioned within the range of a health-related understanding of sustainability: As was discussed in the article by Fischer [9], the notion of sustainability may implicitly assume a more exclusive or inclusive understanding in terms of the dialectic basic ideas. Thus, the application of sustainability could be specified in terms of the extent to which a more exclusive or more inclusive understanding of the notion is applied within the range of the dialectic poles.
- *(4) Transparency, Evaluation, and Policy Design*: Apart from analyzing the respective understanding of sustainability, it would be considered as equally important to be transparent and to communicate the results of the analysis to the public. This would not only meet one of the basic values of sustainability on a general level (i.e., transparency; see [6]), but as well close the circle and afford the opportunity to feed the respective expertise and experience back to the efforts concerning education and awareness-raising. Furthermore, the development of the indicators should be observed and evaluated in terms of their potential to contribute to a more sustainable development of the healthcare system, which might also result in adjustments of concrete health policies. For organizing these adjustments in a participatory manner, design thinking could again be considered a useful tool.

### 4.2. Limitations and Perspectives for Further Research

It is clear that research, whose outcomes are only described in a limited number of articles, needs to focus on certain parts of a given topic. While the specific limitations of each perspective have already been discussed in the respective articles, this section will complementarily outline the general limitations of our research.

First of all, our approach was framed as qualitative research, with a specific focus on the German healthcare system and German health politics. Additionally, we focused on the regulatory sector of the healthcare system, as it observed mainly the political actions of the healthcare stakeholders. Consequently, the other essential constituents of the healthcare system, in particular the financing and the provision of healthcare services [76], were only covered as a side note in order to substantiate the arguments. In a similar way, as stated earlier, the analysis targeted mainly the macro-level of health politics, meaning that the micro- and meso-level did not receive particular attention, even though it is clear that the macro-level resonates with the other levels. Further research could be used to dig deeper in terms of several directions. For example, it would be possible to think of the history of the healthcare system as the history of conflicting basic ideas. This interpretation would enable research to pursue an unconventional approach, which could complement existing historical analyses of the German social state [77]. One could discuss which basic idea was the ‘guiding’ idea for distinct health–political struggles, and to what extent each idea influenced certain outcomes at a particular time in the past, thus leading health policy in a given direction. Additionally, one could think of having a closer look upon the existence or dominance of one of the basic ideas, not only on the macro-level of the regulatory sector of the healthcare system, but also on the meso- and micro-level, or in the financial and provisional sector of the healthcare system. Apart from research aiming to dig deeper, further research could be imagined to build width. This could, for example, happen by ideating and prototyping further design thinking sketches concerning the future shape of the healthcare system.

Another general limitation refers to the conceptual focus of our research on social sustainability. Because of this focus, the ecological pillar of sustainability was admittedly not particularly addressed (even though one could argue that an understanding of sustainability according to the inclusive basic idea would imply the inclusion of environmental factors as well). It is to be noted that alternative approaches in the literature on healthcare sustainability provide for closer insights and more detailed analyses concerning this important matter (e.g., [27,78]).

### 4.3. Practical Implications

How could the practical implications of the three perspectives be summarized? First of all, the framework consisting of five decisive factors of sustainability in healthcare systems [6] could offer orientation for health politicians. They could assess based on the factors, which elements of the healthcare system (e.g., prevention, broad institutionalization of environmental factors) should be strengthened in order to make the healthcare system ‘fit for the future’. It may be noteworthy that only shortly after the article by Fischer [6] had been published, a national Prevention Act was passed—in the aftermath of many unsuccessful previous attempts. As the Act aims to take also broader determinants of health into account, inter alia, by promoting and strengthening measurements of the so-called ‘indirect prevention’ [21], one could argue that some of the suggestions of Fischer [6] have already been implemented in the course of real health policies.

The dialectic consideration of the healthcare system and of health politics, which was presented as the second perspective, may help to differentiate which understanding of sustainability is applied in political discussions. One idea of stimulating this would consist of the formation of an independent ‘watchdog’ organization in the healthcare system. This watchdog organization could be composed of diverse stakeholders of the healthcare system and critically observe health–political actions. Considering the complex trade-off situations between economic and social imperatives, such an organization is currently being requested by actual healthcare stakeholders [79]. Furthermore, one might consider the plans in the coalition treaty of the recently constituted German government between the Christian Democratic Union and the Social Democrats [80], for constituting a commission regarding the harmonization of the different fee structures of doctors (treating their patients either with a statutory or a private health insurance), as well as the intended measures to bridge the differences between the inpatient and outpatient sectors of the German healthcare system of payment, as steps towards the right direction. For practical policymaking, the dialectic of the two basic ideas provides a reasonable explanation for the difficulties in reaching ‘sustainability’ in the healthcare system. Sustainability seems far too complex for just ‘switching on’ the age of sustainability. The described dialectic suggests considering social and economic or inclusive and exclusive imperatives as deeply rooted expressions of interdependent contradictions in the current socio-economic structures of Western societies. In light of this, the suggestion of the second perspective to consider sustainability as a means to look for improvements within the existing socio-economic structures seems legitimate. However, especially the decline of natural resources—both in Western economies and on a global scale—requires fundamentally reassessing the current standards at all levels.

The vision of the VR Health Arena could be seen as a first example of creative approaches to redesigning and complementing the existing (health) political structures. However, the VR Health Arena could only be presented as a first conceptual outline in the course of the research. Its actual implementation would be reserved for a professional production company, which would be in the position of amending the basic concept of the VR Health Arena by making use of its professional expertise and know-how. Research on entertainment–education in the context of sustainability has suggested that forms of cooperation in terms of innovative entertainment concepts carry the potential for mutual benefits for both the entertainment industry and the respective agent with the background in education [63]. Apart from the narrow view of the VR Health Arena, our application of design thinking contributed to scientific progress in terms of the methodological base of (health-related) sustainability science. Because of this successful pilot, one could now think of a more ambitious application of the method in a wider context and with the inclusion of broader parts of the population. Design thinking could then not only be applied as a method, but additionally as a tool to make health–political topics explicitly accessible for broader parts of the society. When speaking of such an extension of the method on a meta-level, the understanding of ‘polity’ could be complemented by an action-oriented, participatory, and transformative notion, which would go beyond the conventional description of political structures.

## Figures and Tables

**Table 1:** The narrative brackets highlighting the internal structure of our research (own illustration).

		First Narrative Bracket: The Three Dimensions of the Political Term		
		Policy	Politics	Polity
**Second narrative bracket: Increasing methodological innovativeness**	**Literature review**	[6]		
	**Grounded theory methodology**		[9,10]	
	**Design Thinking**			[11,12,13]
